# Dietary soy protein reverses obesity-induced liver steatosis and alters fecal microbial composition independent of isoflavone level

**DOI:** 10.3389/fnut.2024.1487859

**Published:** 2024-10-28

**Authors:** Reza Hakkak, Soheila Korourian, Wei Li, Beverly Spray, Nathan C. Twaddle, Christopher E. Randolph, Elisabet Børsheim, Michael S. Robeson II

**Affiliations:** ^1^Department of Dietetics and Nutrition, University of Arkansas for Medical Sciences, Little Rock, AR, United States; ^2^Arkansas Children’s Research Institute, Little Rock, AR, United States; ^3^Department of Pediatrics, University of Arkansas for Medical Sciences, Little Rock, AR, United States; ^4^Department of Pathology, University of Arkansas for Medical Sciences, Little Rock, AR, United States; ^5^Division of Biostatistics Core, Arkansas Children’s Research Institute, Little Rock, AR, United States; ^6^Division of Biochemical Toxicology, National Center for Toxicological Research, US Food and Drug Administration, Jefferson, AR, United States; ^7^Arkansas Children’s Nutrition Center, Little Rock, AR, United States; ^8^Department of Biomedical Informatics, University of Arkansas for Medical Sciences, Little Rock, AR, United States

**Keywords:** obesity, metabolic dysfunction-associated steatotic liver disease, soy protein, isoflavone, microbiota, Zucker rats

## Abstract

**Introduction:**

Metabolic dysfunction-associated steatotic liver disease (MASLD) is a major public health concern that is exacerbated by the obesity pandemic. Dietary interventions have the potential to alleviate obesity-associated MASLD through variable mechanisms, including optimizing the gut microbiota. Previously, we reported that soy protein concentrate (SPC) with low or high levels of isoflavone (LIF or HIF) protected young obese Zucker rats from developing liver steatosis. The current study was designed to test whether SPC-LIF and SPC-HIF diets would reverse liver steatosis and alter fecal microbial composition in adult obese Zucker rats with existing steatosis.

**Methods:**

Six-week-old male obese Zucker rats (*n* = 26) were fed a casein control diet (CAS) for 8 weeks and 7 rats were randomly selected and sacrificed to confirm liver steatosis. The remaining rats were randomly assigned to receive CAS, SPC-LIF, or SPC-HIF diet (*n* = 6–7/group) for an additional 10 weeks.

**Results:**

Compared to CAS diet, feeding SPC-LIF and SPC-HIF diets resulted in significantly lower liver weight, liver steatosis score, and liver microvesicular score (*p* < 0.05), but did not lead to difference in body weight, liver macrovesicular score, serum ALT, or serum AST. Isoflavone levels (e.g., LIF vs. HIF) did not affect any of these measurements except in the SPC-HIF group, which had an additional decrease in liver weight (*p* < 0.05) compared to the SPC-LIF group. The SPC-HIF group also had significantly higher levels of the aglycone forms of daidzein, genistein, and equol as well as the total levels of daidzein, genistein, and equol compared to SPC-LIF or CAS diet fed rats (*p* < 0.05). The distribution of microbial communities based on measures of beta diversity of both SPC-LIF and SPC-HIF groups were significantly different to that of the CAS group (*p* ≤ 0.005). Alpha-diversity did not differ between any of the groups.

**Conclusion:**

Taken together, dietary soy protein can reverse liver steatosis in adult Zucker rats, and the reversal of steatosis is accompanied by alterations in gut microbial composition.

## 1 Introduction

Metabolic dysfunction-associated steatotic liver disease (MASLD) [previously known as non-alcoholic fatty liver disease (NAFLD)] is the most common cause of liver disease in the US and worldwide, affecting about 85 million Americans and about 25% of the global population (1, 2). MASLD is characterized by the excessive accumulation of lipids in the liver and may manifest as simple steatosis or progress to non-alcoholic steatohepatitis (NASH), cirrhosis, and hepatocellular carcinoma (HCC) (3). MASLD does not affect the liver alone. For example, MASLD is an independent risk factor for cardiovascular diseases (CVD) (4, 5). The risk of developing type 2 diabetes is more than 2-fold higher in individuals with MASLD (6).

The cause of MASLD is multi-factorial and varies between individuals. Common contributing factors include genetics, obesity, insulin resistance, and dyslipidemia (7). Obesity is a major risk factor for MASLD in both children and adults (8). According to the 2024 data published by the Centers for Disease Control and Prevention, 2 in 5 adults and 1 in 5 children in the US have obesity (9). Recent data also indicate that the coronavirus disease 2019 (COVID-19) pandemic has worsened obesity rates in US since early 2020 (10, 11).

Obesity promotes MASLD through various inter-connected mechanisms. Obesity often leads to ectopic fat accumulation, defined as the presence of excessive lipid deposition in locations not classically associated with adipose tissue storage (12). The ectopic fat accumulation in the liver (liver steatosis) is the direct cause of MASLD. The ectopic liver lipids, such as diacylglycerol, promotes insulin resistance and inflammation; both are important risk factors for type 2 diabetes and other obesity-associated comorbidities, such as cardiovascular diseases (13). Insulin resistance and heightened inflammation can together form a viscous cycle with liver steatosis and further exacerbate MASLD. Insulin resistance and inflammation both play an etiological role in the development of MASLD and its progression to NASH, cirrhosis, and HCC (14–16).

In addition to the classic metabolic dysregulations, the composition of gut microbiota also plays an important role in the development of MASLD (17). Commensal gut bacteria exist in a symbiotic relationship with the host and are important in the digestion of dietary components and the metabolism of drugs, xenobiotics, and nutrients (18). The microbiota not only produce short-chain fatty acids, vitamins, and other essential nutrients that are absorbed by the host but also compete with pathogenic bacteria for colonization of the GI tract. Intestinal microbiota are essential in mucosal and systemic immunity and can affect energy metabolism and insulin sensitivity in human subjects with metabolic syndrome (19–21). Microbiota-derived molecules, such as lipopolysaccharides (LPS), bacterial capsule carbohydrates, and other endotoxins, are an important source of inflammation to the host (22). The passage of microbiota-derived LPS into the liver and systemic circulation has been implicated in the etiology of a plethora of chronic diseases including MASLD (23–26). The specific population of organisms comprising of intestinal microbiota in an individual is relatively stable, but factors such as diet, disease status, and antimicrobial use can change the composition of bacterial groups, thereby affecting the metabolic capacity of the total microbiota and the health of the host (27, 28). The connections between gut microbiota dysbiosis and diseases, particularly obesity-associated comorbidities including MASLD have been supported by a vast body of research in recent years (17, 29–32).

There are very limited pharmaceutical options to treat MASLD. Easing MASLD disease burden primarily relies on lifestyle modifications, particularly dietary interventions. Studies in both animals and humans suggest that consuming soy protein can protect against dyslipidemia and liver steatosis (33–41). The high-quality soy protein and soy isoflavones are important contributors to the health benefits of soy and soy foods in reducing the risk of chronic diseases (42–44). Soy isoflavones are mainly daidzin and genistin in their glycoside forms with a small amount as aglycone forms (45, 46). Although isoflavones generally have low bioavailability and are metabolized by intestinal commensal bacteria and host cells, they can be detected in blood after consumption (47). Equol is an isoflavone metabolite that is formed from intestinal bacteria metabolizing daidzein. Only 25–50% of the human population are equol producers while all the animal species tested can metabolize daidzein to equol (47).

Previously, we reported that feeding young (7-week-old) obese Zucker rats soy protein concentrate with low or high isoflavones can reduce liver steatosis, liver inflammation, and systemic inflammation (33, 48, 49), suggesting that soy protein may prevent the onset of MASLD when the consumption starts early in life. Both glycoside and aglycone forms of soy isoflavones and equol were detected in sera of rats fed soy protein concentrate with low or high isoflavones, with the concentrations higher in the high isoflavones group (33, 50). However, it is not clear whether dietary soy protein with low- or high-isoflavone can provide similar benefits in reversing existing liver steatosis in adults obese Zucker rats. Furthermore, there are limited reports on the effect of dietary soy protein and isoflavones on gut microbiota. Animal studies suggest that soy protein increases gut microbiota diversity and affect the abundance of specific microbes (51). A limited number of publications also indicate that dietary isoflavones can increase gut microbiota diversity and reduce lipopolysaccharide biosynthesis (52, 53). However, there is no agreement on specific changes of gut microbiota in response soy protein and/or isoflavone consumption. We hypothesized that feeding soy protein concentrate to adult obese Zucker rats will reduce liver steatosis, and alter gut microbial communities compared to a casein control diet. We further expected that the isoflavone levels would have an effect on these changes.

## 2 Materials and methods

### 2.1 Ethics statement

The animal care protocol and procedures in the study was approved by the University of Arkansas for Medical Sciences and Arkansas Children’s Research Institute Institutional Animal Care and Use Committee (Protocol code no. 3968; approved on December 20, 2019) and followed the guidelines of the United States Department of Agriculture (USDA, Washington, DC, USA) Animal Welfare Act.

### 2.2 Experimental design

A total of 26 six-week-old male obese (fa/fa) Zucker rats were purchased from Charles River Laboratories (Wilmington, MA, USA). All rats were fed a semi-purified diet similar to the AIN-93G diet with casein as the protein source (CAS control diet) for 8 weeks. After the initial feeding period, 7 rats were randomly selected and sacrificed to confirm the existence of liver steatosis. The remaining 19 rats were randomly assigned to three dietary groups (6–7 rats per group) and fed a diet with soy protein concentrate containing low isoflavone (SPC-LIF) (*n* = 6), a diet with soy protein concentrate containing high isoflavone (SPC-HIF) (*n* = 6), or the CAS control diet (*n* = 7) for an additional 10 weeks. L-cystine was added at 3 g/kg to the casein diet and 1.2 g/kg to SPC-LIF and SPC-HIF diets and L-methionine was added at 2.2 g/kg to SPC-LIF and SPC-HIF diets to balance the amino acid profile. The SPC-LIF diet had 0.154 mg isoflavone/g protein with an aglycone component of approximately 0.16 mg/g protein. The SPC-HIF diet had 2.153 mg isoflavone/g protein, with an aglycone component of approximately 1.72 mg/g protein. Isoflavone levels were below detection in the CAS diet. Diets were manufactured by Archer Daniels Midland (ADM) (Decatur, IL, USA) with detailed composition reported previously (33). At the end of the experiment, the rats were anesthetized with carbon dioxide and euthanized by decapitation. Livers were removed by dissection without including visceral adipose tissue. Liver weights were measured and recorded. Liver samples from the same lobe of all animals were fixed in 10% buffered formalin for histopathological analysis. Sera were collected by centrifuging blood samples at 4°C and stored at −80°C for subsequent AST and ALT analyses along with isoflavone measurements.

### 2.3 Liver histology

Liver samples fixed in 10% buffered formalin were paraffin-embedded using a Tissue Tek VIP 6 AI Tissue Processor and a Tissue-Tek Embedding Center (Sakura Finetek, Torrance, CA, USA). Tissue blocks were cut on a Microm HM325 microtome (ThermoFisher Scientific, Waltham, MA), and stained with hematoxylin and eosin (H&E) for examination by a pathologist using a blinded protocol. Liver sections were evaluated for microvesicular and macrovesicular steatosis. Steatosis within hepatocytes was semi-quantitated using the following scoring criteria as we used in previous studies (54): (0) no steatosis; (1) < 25%; (2) 25–50%; (3) 50–75%; and (4) > 75%.

### 2.4 Serum ALT and AST

Serum ALT and AST were determined using a COBAS INGEGRA^®^ 400 Plus chemistry analyzer (Roche Diagnostics Corp. Indianapolis, IN) according to the manufacturer’s instructions (33).

### 2.5 Measurement of serum isoflavones and metabolites

Both the aglycone forms of genistein, daidzein, and equol as well as the total levels of genistein, daidzein, and equol were determined in sera using a validated method of supported liquid extraction and UPLC-ESI/MS/MS (55), with a Xevo TQ-S mass spectrometer (Waters, Inc., Milford, MA). Briefly, sera were diluted and mixed with stable-labeled internal standards for supported liquid extraction. Samples were evaporated, reconstituted, and analyzed using UPLC-ESI/MS/MS. For total isoflavone measurements, samples were enzymatically deconjugated prior to extraction (50). The limit of quantification (LOQ) and limit of detection (LOD) were established using signal-to-noise ratios of 10 and 3, respectively. Using these criteria and a sample volume of 70 μL, the LOQ for aglycone analysis of genistein, daidzein, and equol are roughly 0.001, 0.0006, and 0.001 μM, respectively. For analyses of total genistein, daidzein, and equol using 10 μL serum, the LOQ for genistein, daidzein, and equol are roughly 0.02, 0.02, and 0.02 μM, respectively. The LOD of each analyte is roughly 1/3 of the LOQ.

### 2.6 Fecal DNA extraction and sequencing

Total microbial DNA was collected directly from the fecal samples using a PowerSoil^®^ DNA isolation kit (MoBio Laboratories, Inc., Carlsbad, CA). Isolated DNA was used for amplifying the V4 region of 16S rRNA gene as per Kozich et al. (56). Data were collected using the Illumina NextSeq 2000. MIMARKS (57) compliant data are available from the Sequence Read Archive (SRA) at the National Center for Biotechnology Information (NCBI), under BioProject PRJNA1152982.

### 2.7 Amplicon sequencing and analyses

QIIME 2 (58) was used to process and analyze the amplicon sequence data, any commands prefixed by “q2” are QIIME 2 plugins. Demultiplexed paired-end reads were imported via a QIIME 2 manifest file. The V4 primers were removed via q2-cutadapt (59), and subsequently denoised with q2-dada2 (60), using the following settings: forward truncation length 250, reverse truncation length 222, using the pooling method “pseudo” and the “pooled” chimera method, in conjunction with the minimum fold parent over abundance value of 8. RESCRIPt was used to prepare a Naïve Bayes classifier for the V4 hyper-variable region from the SILVA 138.1 reference database and used to classify the resulting exact sequence variants (ESVs) / features with q2-feature-classifier (61–66). Taxonomy based filtering was performed with q2-taxa filter-table to remove any ESVs that were classified as “chloroplast,” “mitochondria,” “eukaryota,” and “unclassified.” Also, any features that did not have at least a phylum-level classification were also removed. ESVs that did not have at least a 90% identity and query alignment to the SILVA reference database were removed with q2-quality-control. ESVs were inserted into a SILVA reference tree using q2-fragment-insertion (67). The feature-table was subsequently filtered using feature-table filter-features-conditionally by removing any features that did not appear in at least ∼10.5% of all samples (2 samples) and accounted for less than a 0.01% total abundance within those samples. Diversity analysis was run via q2-diversity core-metrics-phylogenetic with data rarefied to 75,000 reads per sample. Differential abundance analysis was performed using ANCOM-BC (68) and ALDEx2 (69, 70) on non-rarefied data. Plots were made using QIIME 2 and dokdo (58, 71).

### 2.8 Statistical analysis

Data on each dependent variable were assessed for normality using histograms and the Shapiro-Wilk test. Equal variances among the diet groups were assessed with Levene’s test. To determine if any of the dependent variables differed significantly between dietary groups, a general linear model procedure was employed with treatment as the main effect. If a significant main effect of treatment was noted, post-hoc tests were computed. *P*-values from multiple comparisons were adjusted using Holm’s procedure. *P* < 0.05 was considered statistically significant. All analyses were computed with SAS software, version 9.4 (SAS Institute Inc., Cary NC, USA).

## 3 Results

### 3.1 Body weight, liver weight, liver histopathology, and serum aminotransferase levels

**FIGURE 1:**
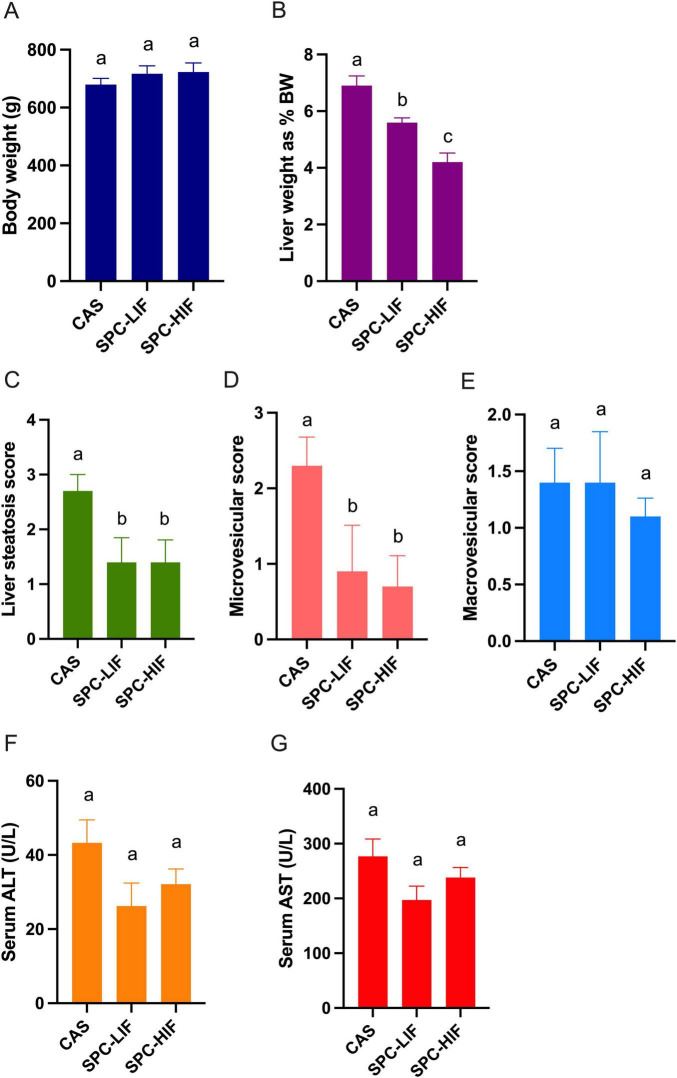
Body weight **(A)**, liver weight as percent body weight **(B)**, liver histopathological scores **(C,D,E)**, serum aspartate aminotransferase (AST) **(F)**, and alanine aminotransferase (ALT) **(G)** levels in obese Zucker rats fed casein control (CAS), soy protein concentrate with low isoflavone (SPC-LIF), or soy protein concentrate with high isoflavone (SPC-HIF) diet. Data are expressed as mean ± S.E. Means labeled with different superscripts are significantly different from each other in the same graph (*p* < 0.05).

**FIGURE 2:**
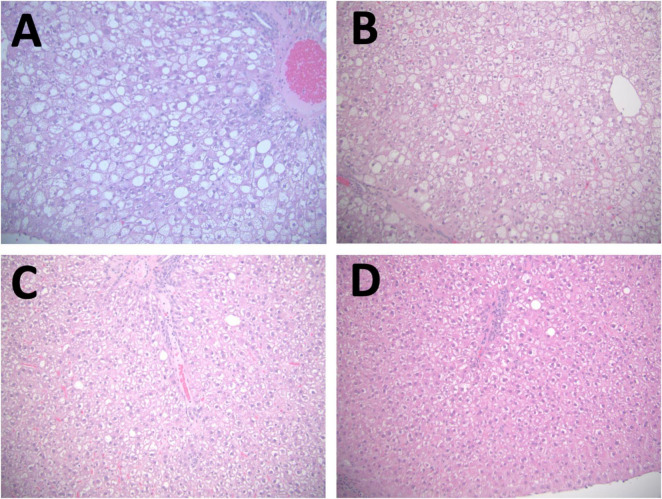
Representative images of hematoxylin and eosin-stained liver sections. A representative image is shown from baseline CAS 8 weeks **(A)**, the CAS (18 weeks) group **(B)**, the SPC-LIF group **(C)**, and the SPC-HIF group **(D)**. All images are representing both peripheral and central regions at magnifications are 20×.

After all rats were fed CAS diet for 8 weeks, baseline liver steatosis was confirmed in the 7 rats that were randomly chosen and sacrificed. For the remaining 19 rats, they were randomly assigned to three groups and fed the CAS (*n* = 7), SPC-LIF (*n* = 6), or SPC-HIF (*n* = 6) diet for an additional 10 weeks. At week 18, body weight (Figure 1A), liver macrovesicular score (Figure 1E), serum ALT (Figure 1F), and serum AST (Figure 1G) did not differ between any dietary groups. Liver weight as percent bodyweight (Figure 1B), liver steatosis score (Figure 1C), and liver microvesicular score (Figure 1D) were significantly lower in the SPC-LIF and SPC-HIF groups compared to the CAS group. Isoflavone level did not affect liver steatosis score or liver microvesicular score (Figures 1D, E). In contrast, liver weights as percent body weight were significantly lower in the SPC-HIF group compared to the SPC-LIF group (Figure 1B). Representative images of hematoxylin and eosin-stained liver sections from the baseline and after feeding CAS, SPC-LIF, or SPC-HIF diet for 10 weeks are shown in Figure 2.

The group at the end of the experiment after fed the CAS diet for a total of 18 weeks had comparable liver histopathological scores with those of the group sacrificed at baseline after 8 weeks on the CAS diet (Supplementary Table 1). The only differences were the expected differences in body weights, and lower ALT levels after 18 weeks (Supplementary Table 1). Taken together, feeding the CAS diet to obese Zucker rats for 8 weeks led to well-established liver steatosis that was maintained for the duration of the study.

### 3.2 Serum isoflavone levels

**FIGURE 3:**
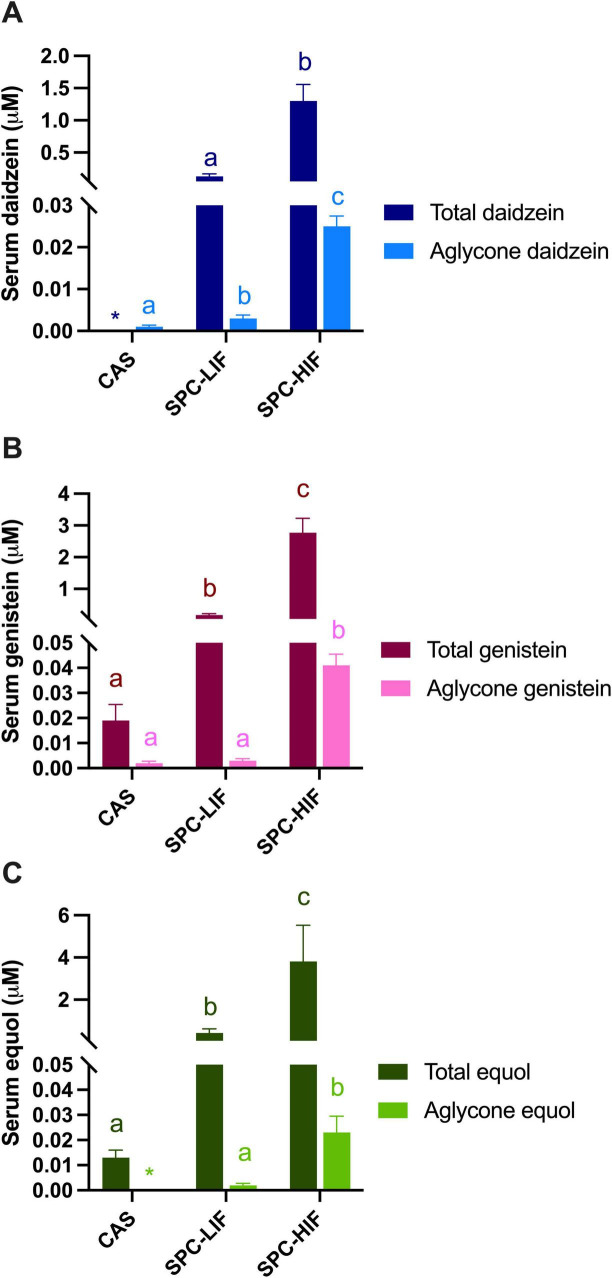
Serum total and aglycone forms of daidzein **(A)**, genistein **(B)**, and equol **(C)** levels in obese Zucker rats fed casein control (CAS), soy protein concentrate with low isoflavone (SPC-LIF), or soy protein concentrate with high isoflavone (SPC-HIF) diet. Data are expressed as mean ± S.E. Means labeled with different superscripts are significantly different from each other in the same colored group (*p* < 0.05). *Only one rat had detectable values of aglycone equol and total daidzein.

After rats were fed the CAS, SPC-LIF, or SPC-HIF diet, levels of different isoflavones were determined in sera. SPC-HIF diet-fed rats had significantly higher levels of all aglycone forms of and total daidzein, genistein, and equol compared to SPC-LIF or CAS diet fed rats (Figures 3A–C). SPC-LIF diet-fed rats had significantly higher serum levels of aglycone daidzein, aglycone equol, total daidzein, total genistein, and total equol compared to CAS diet fed rats (Figures 3A–C).

### 3.3 Gut microbiota

**FIGURE 4:**
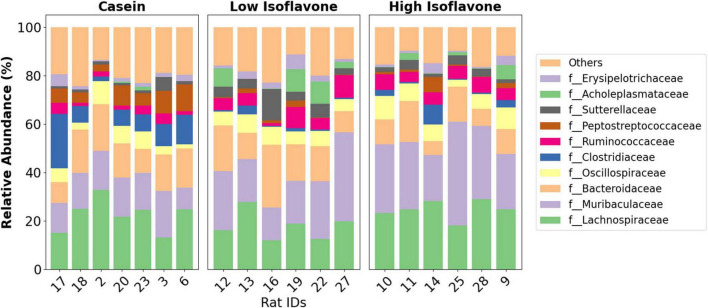
Taxonomy. Family-level taxonomic distribution of gut microbiota by the treatment groups: casein, low isoflavone, high isoflavone.

**FIGURE 5:**
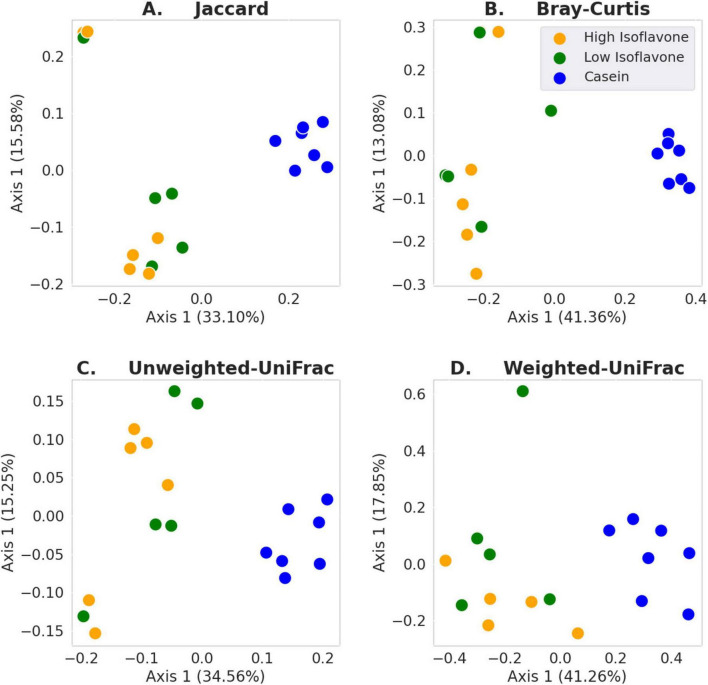
Beta diversity. **(A)** Jaccard: LIF or HIF vs. Casein *p* ≤ 0.003; LIF vs. HIF *p* ≥ 0.211. **(B)** Bray-Curtis: LIF or HIF vs. Casein *p* ≤ 0.003; LIF vs. HIF *p* ≥ 0.290. **(C)** Unweighted-UniFrac: LIF or HIF vs. Casein *p* ≤ 0.003; LIF vs. HIF *p* ≥ 0.173. **(D)** Weighted-UniFrac: LIF or HIF vs. Casein *p* ≤ 0.005; LIF vs. HIF *p* ≥ 0.299.

**FIGURE 6:**
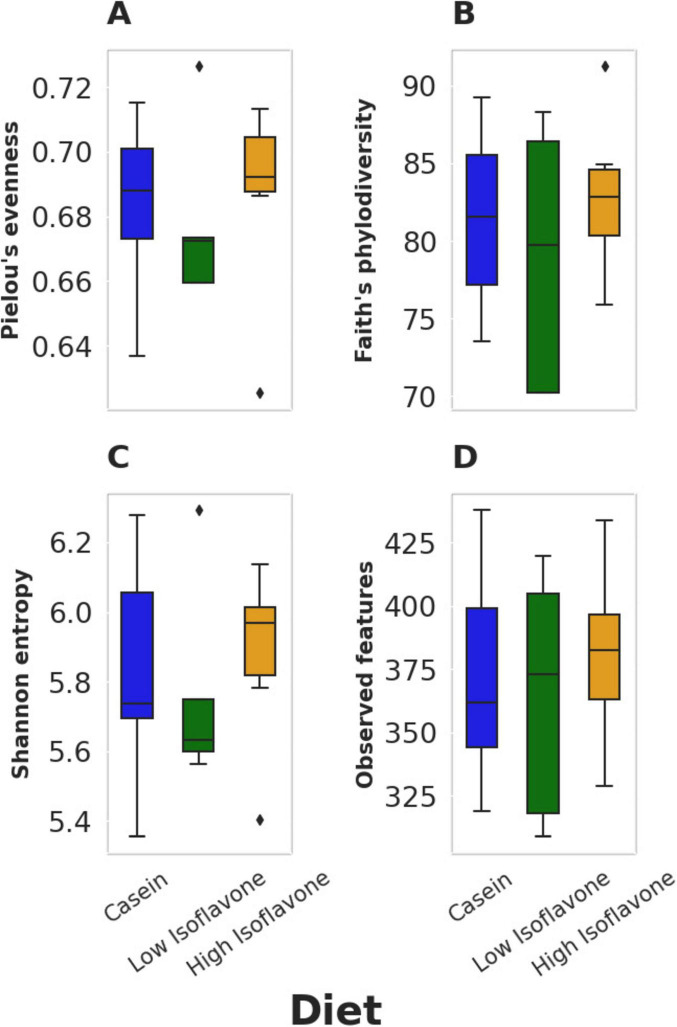
Alpha diversity. **(A)** Pielou’s evenness: *p* ≥ 0.668. **(B)** Faith’s phylodiversity: *p* ≥ 0.668. **(C)** Shannon entropy: *p* ≥ 0.668. **(D)** observed features: *p* ≥ 0.775.

The distribution of microbial communities (Figure 4) of both LIF and HIF treated rats were significantly different compared to the casein treated rats, based on all measures of beta diversity (Figure 5). However, no significant differences were observed between LIF and HIF. Conversely, no differences in alpha diversity were observed between any of the treatments (Figure 6).

Differential abundance of microbiota was ascertained by ANCOM-BC and ALDEx2. Only those features which were detected by both approaches were considered (Supplementary Figure 1). A total of 44 microbial features were found to be differentially abundant between LIF and HIF vs. Casein. There were no differentially enriched microbes between LIF and HIF. The microbial features enriched within either isoflavone treatment were as follows: two features within the *Muribaculaceae*, six features within the *Lachnospiraceae*, three features within the *Oscillospiraceae*, and one feature from within the *[Eubacterium] coprostanoligenes* group.

Microbial features enriched in the casein were as follows: one feature in *Eggerthellaceae*, one feature in *Bacteroidaceae*, one feature in *Deferribacteraceae*, one feature in *Streptococcaceae*, two features within *Clostridiaceae*, sixteen features within *Lachnospiraceae*, seven features within *Oscillospiraceae*, two features within *Ruminococcaceae*, and one feature in *Peptostreptococcaceae*.

## 4 Discussion

Previously, we reported that feeding 7-week-old young obese Zucker rats a SPC-LIF or SPC-HIF diet for 9 or 18 weeks led to significantly reduced liver steatosis compared to the CAS diet (33, 50). Results from the current study demonstrate that the anti-steatotic effect of dietary soy protein is not limited to young Zucker rats prior to the onset of liver steatosis. When SPC-LIF or SPC-HIF diets were fed for 10 weeks to adult obese Zucker rats with existing liver steatosis, SPC diets were able to reverse liver steatosis regardless of their isoflavone content. Like our previous observations in young Zucker rats, the benefit of SPC diets was independent from body weight change, as body weights did not differ between any of the CAS, SPC-LIF, and SPC-HIF groups at the end of the feeding period (Figure 1A).

Soy protein has anti-inflammatory properties (72, 73) that may mediate its benefit in reducing the risk of chronic diseases including MASLD. The contribution of inflammation to the development of MASLD is supported by the resistance to liver steatosis in TNF-α^–/–^ mice and mice treated with a TNF-α receptor antagonist (74, 75). Indeed, we reported that feeding SPC-LIF or SPC-HIF diet to both young and adult obese Zucker rats reduced the expression of inflammatory genes including TNF-α, MCP-1, and iNOS in the liver (49, 76). In individuals with obesity, inflammation arises from two main sources—the dysfunctional adipose tissue (77) and the gut microbiota (60), with the latter particularly relevant to the etiology of MASLD due to the gut-liver axis—a bidirectional relationship between the gut and its microbiota, and the liver (78). Obesity is associated with increased passage of gut bacteria-derived inflammatory molecules, such as LPS, to the liver via the hepatic portal vein, then into the systemic circulation (60, 79). Gut bacteria-derived LPS have been implicated in the etiology of obesity-associated chronic diseases, including heart disease, stroke, cancer, diabetes, chronic kidney disease, MASLD, and autoimmune and neurodegenerative conditions (23–26). Patients diagnosed with MASLD have elevated LPS levels in circulation compared to healthy controls (80). LPS levels in blood and liver biopsy samples correlate with the severity of disease in patients with MASLD (81). Animal studies have demonstrated a causal relationship between LPS exposure and MASLD. In obese (*fa/fa*) Zucker rats, intraperitoneal LPS injection exacerbates hepatic steatosis (82). Low-dose subcutaneous LPS injection worsens high fat diet induced MASLD in mice (83). Consistent with these reports, we showed that feeding both young and adult obese Zucker rats SPC-LIF or SPC-HIF diet decreased the expression of LPS-binding protein, a marker for LPS exposure in the liver (49, 76), suggesting that liver steatosis in obese Zucker rats may be exacerbated by the inflammatory stimuli arising from the GI tract, which can be attenuated by SPC. In addition to the liver inflammatory makers, we have reported significant differences in the expression of genes involved in hepatic lipid metabolism and lipid transport between soy protein-fed and casein-fed obese Zucker rats (84, 85). Counterintuitively, feeding soy protein significantly increased liver expression of lipogenic genes including fatty acid synthase (FASN), malic enzyme 1 (ME1), 6-phosphogluconate dehydrogenase (6PGD), Sterol Regulatory Element Binding Protein-1c (SREBP-1c) and SREBP-2 genes in the livers of obese rats, suggesting soy protein reduces liver steatosis not by inhibiting lipogenesis (84). Soy protein significantly increased the expression of acyl-CoA oxidase 1 (ACOX1) in the liver of obese Zucker rats (85). ACOX1 catalyzes the initial step of the peroxisomal beta-oxidation of very long fatty acids, suggesting beta-oxidation may be upregulated in soy protein fed obese rats. Pathway analyses from the same study also detected an upregulation of pathways involved in lipid transport/export in soy protein-fed obese Zucker rats (85). Taken together, decreased inflammation, increased beta-oxidation of long-chain fatty acids, and increase export of lipid from the liver are likely to explain the reduced liver steatosis in soy protein fed obese rats.

Gut microbial composition is important for the mediation of inflammatory stimuli from the gut to the liver. The composition of gut microbes can determine the amount and type of bacteria-derived pro-inflammatory molecules, thereby shaping the origin of the inflammatory stimuli from the GI tract. Furthermore, gut microbiota can interact with intestinal cells and modulate GI tract permeability (86–88). Obesity is associated with profound changes in gut microbiota composition and increases GI tract permeability (89–91). Because the reversal of liver steatosis in obese Zucker rats by SPC-LIF and SPC-HIF diets may be due to decreased inflammatory stimuli from the gut, we characterized the effect of SPC-LIF and SPC-HIF diets on gut microbiota composition in these adult Zucker rats.

Gut microbiota composition was strongly associated with soy protein concentrate treatment (either high or low isoflavone) vs. casein (Figure 5). No significant difference was observed between the high and low dose isoflavone treatments themselves. A possible explanation for the lack of differences between the two isoflavone groups, may be limited sampling (Figures 5, 6).

Differential abundance analyses, using exact sequence variants, revealed that we could differentiate closely related microbial features that would have otherwise been lost if we clustered the sequences into OTUs or by taxonomy, (Supplementary Figure 1) (65, 92). That is, features within the same genus displayed different distributions among the treatment groups (Supplementary Figure 1). Like prior studies (52, 53), we also observed differentially enriched microbial taxa due to soy protein concentrate treatments (Supplementary Figure 1). For example, we observed that features belonging to the *Muribaculaceae* (panels 3, 4; Low and High Isoflavone) and the *Eubacterium coprostanoligenes* (panel 43; High Isoflavone) groups, were enriched in the soy protein concentrate treatment groups. The *Muribaculaceae* are common murine microbes that can generate short-chain fatty acids and play a role in intestinal barrier function (93), however, others have shown some members of the *Muribaculaceae* can be proinflammatory (94). The *Eubacterium coprostanoligenes* group is noteworthy, as it has been shown to reduce intestinal mucositis complications due to chemotherapy and enhance the intestinal mucus barrier (95). *E. coprostanoligenes* has also been shown to metabolize cholesterol, potentially contributing to the reduced risk of cardiovascular disease by decreasing serum cholesterol levels (96, 97). Further research is required to determine if *E. coprostanoligenes* is performing similar roles here.

A limitation of our study is that while we demonstrated an association between the benefit of SPC in reversing liver steatosis and changes in microbial composition, we could not establish that this relationship is causal. The source of dietary protein is known to affect gut microbiota in various species including the rats, with main contributing factors include differences in amino acid composition and amino acid sequence in peptides that may lead to difference in their metabolism/fermentation by the gut bacteria (98, 99). Although we reason that the optimization of microbial composition by SPC is likely the cause of the liver steatosis reversal, due to the bi-directional nature of the gut-liver axis (78), we cannot rule out that reduced liver steatosis may have driven the changes in microbial composition in SPC fed rats. Future studies on the temporal relationship between liver steatosis and gut microbial changes in SPC fed rats and fecal transplant studies using germ-free animals will shed light on the dynamic interaction between SPC-induced changes in the liver and the gut. Another limitation of our study is that although body weight did not significantly differ between CAS, SPC-LIF, and HPC-HIF fed obese rats, there may be differences in adipose tissue distribution (e.g., subcutaneous vs. visceral adipose tissues) between groups that may contribute to differences in liver pathophysiology and fecal microbial composition. We plan to use magnetic-resonance imaging (SRI) and dual-energy x-ray absorptiometry (DEXA) to investigate such possibilities in future studies. The method we used to score liver histopathology focused on microvesicular and macrovesicular steatosis. A limitation of this scoring method was that it did not account for histological features such as lobular inflammation, hepatocellular ballooning, and fibrosis. To address this limitation, we will consider adapting an NAS activity score system (100) as an alternative in future studies. Lastly, although we attribute the benefit of SPC diets mainly to soy protein, SPC-HIF diet did further reduce liver weight/body weight ratio compared to SPC-LIF diet (Figure 1B), suggesting isoflavones in SPC may provide additional benefit that is not enough to further improve liver pathological scores within the duration of the study (Figures 1C–E). Future studies with longer feeding periods or higher isoflavone doses are needed to characterize the potential additional benefits of soy isoflavones in reversing obesity-associated liver steatosis.

## 5 Conclusion

In conclusion, soy protein concentrate compared to casein (control) reverses liver steatosis independent of isoflavone level. Similarly, gut microbial composition differed between casein and the high and low isoflavone dosages. Findings from this study suggest that consuming soy protein may provide health benefits to both the liver and the gut.

## Data Availability

The datasets presented in this study are available from the Sequence Read Archive (SRA) at the National Center for Biotechnology Information (NCBI), under BioProject PRJNA1152982. Further inquiries can be directed to the corresponding authors.

## Supplementary material

Supplementary Figure 1 Differential abundance. ANCOM-BC and ALDEx2 were used to identify differentially abundant microbial features between all three treatment groups. Only features found to be significantly different with both ANCOM-BC and ALDEx2 are shown. There were no significant differences between LIF and HIF, but each isoflavone treatment did contain significantly different features as compared to the casein treatment, mirroring the beta diversity findings. Significance (*q* ≥ 0.05) is presented in the compact letter display format. Where by those categories with the same letter are not significantly different from one another, and those with different letters are significantly different one another.

## References

[1] Mitra S De A Chowdhury A. Epidemiology of non-alcoholic and alcoholic fatty liver diseases. *Transl Gastroenterol Hepatol.* (2019) 5:16. 10.21037/tgh.2019.09.08 PMC7063528 32258520

[2] Shetty A Syn W. Health and economic burden of nonalcoholic fatty liver disease in the United States and its impact on veterans. *Fed Pract.* (2019) 36(1):14–9. 30766413 PMC6366581

[3] Lazarus J Mark H Anstee Q Arab J Batterham R Castera L Advancing the global public health agenda for NAFLD: A consensus statement. *Nat Rev Gastroenterol Hepatol.* (2022) 19(1):60–78. 10.1038/s41575-021-00523-4 34707258

[4] Lu H Liu H Hu F Zou L Luo S Sun L. Independent association between nonalcoholic fatty liver disease and cardiovascular disease: A systematic review and meta-analysis. *Int J Endocrinol.* (2013) 2013:124958. 10.1155/2013/124958 PMC3639649 23690766

[5] Francque S van der Graaff D Kwanten W. Non-alcoholic fatty liver disease and cardiovascular risk: Pathophysiological mechanisms and implications. *J Hepatol.* (2016) 65(2):425–43. 27091791 10.1016/j.jhep.2016.04.005

[6] Gastaldelli A Cusi K. From NASH to diabetes and from diabetes to NASH: Mechanisms and treatment options. *JHEP Rep.* (2019) 1(4):312–28. 32039382 10.1016/j.jhepr.2019.07.002 PMC7001557

[7] Younossi Z Anstee Q Marietti M Hardy T Henry L Eslam M Global burden of NAFLD and NASH: Trends, predictions, risk factors and prevention. *Nat Rev Gastroenterol Hepatol.* (2018) 15(1):11–20. 28930295 10.1038/nrgastro.2017.109

[8] Yu E Schwimmer J. Epidemiology of pediatric nonalcoholic fatty liver disease. *Clin Liver Dis (Hoboken).* (2021) 17(3):196–9. 33868665 10.1002/cld.1027 PMC8043694

[9] Obesity Centers for Disease Control and Prevention. (2024). Available online at: https://www.cdc.gov/obesity/index.html (accessed October 2, 2024).

[10] Restrepo B. Obesity prevalence among U.S. adults during the COVID-19 pandemic. *Am J Preventive Med.* (2022) 63(1):102–6. 10.1016/j.amepre.2022.01.012 PMC8977388 35725124

[11] Lange S Kompaniyets L Freedman D Kraus E Porter R Blanck H Longitudinal trends in body mass index before and during the COVID-19 pandemic among persons aged 2-19 years - United States, 2018-2020. *MMWR Morb Mortal Wkly Rep.* (2021) 70(37):1278–83. 10.15585/mmwr.mm7037a3 34529635 PMC8445379

[12] Britton K Fox C. Ectopic fat depots and cardiovascular disease. *Circulation.* (2011) 124(24):e837–41. 22156000 10.1161/CIRCULATIONAHA.111.077602

[13] Byrne C Targher G. Ectopic fat, insulin resistance, and nonalcoholic fatty liver disease. *Arteriosc Thrombosis Vascular Biol.* (2014) 34(6):1155–61. 10.1161/ATVBAHA.114.303034 24743428

[14] Nogueira J Cusi K. Role of insulin resistance in the development of nonalcoholic fatty liver disease in people with type 2 diabetes: From bench to patient care. *Diabetes Spect.* (2024) 37(1):20–8. 10.2337/dsi23-0013 38385099 PMC10877218

[15] Petrescu M Vlaicu S Ciumărnean L Milaciu M Mărginean C Florea M Chronic inflammation-A link between nonalcoholic fatty liver disease (NAFLD) and dysfunctional adipose tissue. *Medicina (Kaunas).* (2022) 58(5):641. 10.3390/medicina58050641 PMC9147364 35630058

[16] Luci C Bourinet M Leclère P Anty R Gual P. Chronic inflammation in non-alcoholic steatohepatitis: Molecular mechanisms and therapeutic strategies. *Front Endocrinol.* (2020) 11:597648. 10.3389/fendo.2020.597648 33384662 PMC7771356

[17] Aron-Wisnewsky J Vigliotti C Witjes J Le P Holleboom A Verheij J Gut microbiota and human NAFLD: Disentangling microbial signatures from metabolic disorders. *Nat Rev Gastroenterol Hepatol.* (2020) 17(5):279–97. 10.1038/s41575-020-0269-9 32152478

[18] Ley R Lozupone C Hamady M Knight R Gordon J. Worlds within worlds: Evolution of the vertebrate gut microbiota. *Nat Rev Microbiol.* (2008) 6(10):776–88. 18794915 10.1038/nrmicro1978 PMC2664199

[19] Goldszmid R Trinchieri G. The price of immunity. *Nat Immunol.* (2012) 13(10):932–8. 22990891 10.1038/ni.2422

[20] Maynard C Elson C Hatton R Weaver C. Reciprocal interactions of the intestinal microbiota and immune system. *Nature.* (2012) 489(7415):231–41. 22972296 10.1038/nature11551 PMC4492337

[21] Vrieze A Van Nood E Holleman F Salojärvi J Kootte R Bartelsman J Transfer of intestinal microbiota from lean donors increases insulin sensitivity in individuals with metabolic syndrome. *Gastroenterology.* (2012) 143(4):913-6.e7. 10.1053/j.gastro.2012.06.031 22728514

[22] Al Bander Z Nitert M Mousa A Naderpoor N. The Gut Microbiota and Inflammation: An Overview. *Int J Environ Res Public Health.* (2020) 17(20):7618. 10.3390/ijerph17207618 PMC7589951 33086688

[23] Cani P Amar J Iglesias M Poggi M Knauf C Bastelica D Metabolic endotoxemia initiates obesity and insulin resistance. *Diabetes.* (2007) 56(7):1761–72. 17456850 10.2337/db06-1491

[24] Mitaka C. Clinical laboratory differentiation of infectious versus non-infectious systemic inflammatory response syndrome. *Clin Chim Acta.* (2005) 351(1–2):17–29. 15563869 10.1016/j.cccn.2004.08.018

[25] Fukui H. Increased intestinal permeability and decreased barrier function: Does It really influence the risk of inflammation? *Inflamm Intest Dis.* (2016) 1(3):135–45. 29922669 10.1159/000447252 PMC5988153

[26] Carnevale R Sciarretta S Valenti V di Nonno F Calvieri C Nocella C Low-grade endotoxaemia enhances artery thrombus growth via toll-like receptor 4: Implication for myocardial infarction. *Eur Heart J.* (2020) 41(33):3156–65. 10.1093/eurheartj/ehz893 31898723

[27] Ley R Turnbaugh P Klein S Gordon J. Microbial ecology: Human gut microbes associated with obesity. *Nature.* (2006) 444(7122):1022–3. 17183309 10.1038/4441022a

[28] Turnbaugh P Ridaura V Faith J Rey F Knight R Gordon J. The effect of diet on the human gut microbiome: A metagenomic analysis in humanized gnotobiotic mice. *Sci Transl Med.* (2009) 1(6):6ra14. 10.1126/scitranslmed.3000322 20368178 PMC2894525

[29] Aron-Wisnewsky J Gaborit B Dutour A Clement K. Gut microbiota and non-alcoholic fatty liver disease: New insights. *Clin Microbiol Infect.* (2013) 19(4):338–48. 23452163 10.1111/1469-0691.12140

[30] Wieland A Frank D Harnke B Bambha K. Systematic review: Microbial dysbiosis and nonalcoholic fatty liver disease. *Aliment Pharmacol Ther.* (2015) 42(9):1051–63. 26304302 10.1111/apt.13376

[31] Roychowdhury S Selvakumar P Cresci G. The role of the gut microbiome in nonalcoholic fatty liver disease. *Med Sci.* (2018) 6(2):47. 10.3390/medsci6020047 PMC6024579 29874807

[32] Hrncir T Hrncirova L Kverka M Hromadka R Machova V Trckova E Gut microbiota and NAFLD: Pathogenetic mechanisms, microbiota signatures, and therapeutic interventions. *Microorganisms.* (2021) 9(5):957. 10.3390/microorganisms9050957 33946843 PMC8146698

[33] Hakkak R Spray B Børsheim E Korourian S. Diet containing soy protein concentrate with low and high isoflavones for 9 weeks protects against non-alcoholic fatty liver steatosis using obese zucker rats. *Front Nutr.* (2022) 9:913571. 10.3389/fnut.2022.913571 35811988 PMC9258741

[34] Hakkak R Gauss C Bell A Korourian S. Short-term soy protein isolate feeding prevents liver steatosis and reduces serum ALT and AST levels in obese female zucker rats. *Biomedicines.* (2018) 6(2):55. 10.3390/biomedicines6020055 29757972 PMC6027420

[35] Xiao C Hendry A. Hypolipidemic effects of soy protein and isoflavones in the prevention of non-alcoholic fatty liver disease- a review. *Plant Foods Hum Nutr.* (2022) 77(3):319–28. 10.1007/s11130-022-00984-1 35678936 PMC9463339

[36] Zhang S Kumari S Gu Y Wu X Li X Meng G Soy food intake is inversely associated with newly diagnosed nonalcoholic fatty liver disease in the TCLSIH cohort study. *J Nutr.* (2020) 150(12):3280–7. 10.1093/jn/nxaa297 33097932

[37] Li Y Deng X Guo X Zhang F Wu H Qin X Preclinical and clinical evidence for the treatment of non-alcoholic fatty liver disease with soybean: A systematic review and meta-analysis. *Front Pharmacol.* (2023) 14:1088614. 10.3389/fphar.2023.1088614 36762106 PMC9907442

[38] Anderson J Johnstone B Cook-Newell M. Meta-analysis of the effects of soy protein intake on serum lipids. *N Engl J Med.* (1995) 333(5):276–82. 7596371 10.1056/NEJM199508033330502

[39] Ascencio C Torres N Isoard-Acosta F Gómez-Pérez F Hernández-Pando R Tovar A. Soy protein affects serum insulin and hepatic SREBP-1 mRNA and reduces fatty liver in rats. *J Nutr.* (2004) 134(3):522–9. 10.1093/jn/134.3.522 14988441

[40] Tovar A Murguía F Cruz C Hernández-Pando R Aguilar-Salinas C Pedraza-Chaverri J A soy protein diet alters hepatic lipid metabolism gene expression and reduces serum lipids and renal fibrogenic cytokines in rats with chronic nephrotic syndrome. *J Nutr.* (2002) 132(9):2562–9. 10.1093/jn/132.9.2562 12221209

[41] Zhan S Ho S. Meta-analysis of the effects of soy protein containing isoflavones on the lipid profile. *Am J Clin Nutr.* (2005) 81(2):397–408. 15699227 10.1093/ajcn.81.2.397

[42] Messina M. Soy and health update: evaluation of the clinical and epidemiologic literature. *Nutrients.* (2016) 8(12):754. 10.3390/nu8120754 27886135 PMC5188409

[43] Badger T Ronis M Hakkak R Rowlands J Korourian S. The health consequences of early soy consumption. *J Nutr.* (2002) 132(3):559s–65s. 11880593 10.1093/jn/132.3.559S

[44] Hakkak R Korourian S Shelnutt S Lensing S Ronis M Badger T. Diets containing whey proteins or soy protein isolate protect against 7,12-dimethylbenz(a)anthracene-induced mammary tumors in female rats. *Cancer Epidemiol Biomarkers Prev.* (2000) 9(1):113–7. 10667471

[45] Kalaiselvan V Kalaivani M Vijayakumar A Sureshkumar K Venkateskumar K. Current knowledge and future direction of research on soy isoflavones as a therapeutic agents. *Pharmacogn Rev.* (2010) 4(8):111–7. 10.4103/0973-7847.70900 22228950 PMC3249910

[46] Miadoková E. Isoflavonoids - an overview of their biological activities and potential health benefits. *Interdiscip Toxicol.* (2009) 2(4):211–8. 10.2478/v10102-009-0021-3 21217857 PMC2984117

[47] Mayo B Vázquez L Flórez A. Equol: A bacterial metabolite from the daidzein isoflavone and its presumed beneficial health effects. *Nutrients.* (2019) 11(9):2231. 10.3390/nu11092231 31527435 PMC6770660

[48] Li W Twaddle N Spray B Nounamo B Monzavi-Karbassi B Hakkak R. Feeding soy protein concentrates with low and high isoflavones alters 9 and 18 weeks serum isoflavones and inflammatory protein levels in lean and obese zucker rats. *J Med Food.* (2023) 26(2):120–7. 10.1089/jmf.2022.0100 36720082

[49] Li W Hakkak R. Feeding soy protein concentrates with low or high isoflavone decreases liver inflammation by reducing lipopolysaccharide translocation. *Front Nutr.* (2023) 10:1278158. 10.3389/fnut.2023.1278158 38075211 PMC10699199

[50] Li W Spray B Børsheim E Korourian S Hakkak R. Long-term feeding soy protein concentrates protect against hepatic steatosis independent of isoflavone levels. *J Med Food.* (2023) 26:911–8. 10.1089/jmf.2023.0118 37971778

[51] Belobrajdic D James-Martin G Jones D Tran C. Soy and gastrointestinal health: A review. *Nutrients.* (2023) 15(8):1959. 10.3390/nu15081959 37111176 PMC10144768

[52] Ghimire S Cady N Lehman P Peterson S Shahi S Rashid F Dietary isoflavones alter gut microbiota and lipopolysaccharide biosynthesis to reduce inflammation. *Gut Microbes.* (2022) 14(1):2127446. 10.1080/19490976.2022.2127446 PMC9542810 36179318

[53] Huang L Zheng T Hui H Xie G. Soybean isoflavones modulate gut microbiota to benefit the health weight and metabolism. *Front Cell Infect Microbiol.* (2022) 12:1004765. 10.3389/fcimb.2022.1004765 36118025 PMC9478439

[54] Korourian S Hakkak R Ronis M Shelnutt S Waldron J Ingelman-Sundberg M Diet and risk of ethanol-induced hepatotoxicity: Carbohydrate-fat relationships in rats. *Toxicol Sci.* (1999) 47(1):110–7. 10.1093/toxsci/47.1.110 10048159

[55] Doerge D Woodling K Churchwell M Fleck S Helferich W. Pharmacokinetics of isoflavones from soy infant formula in neonatal and adult rhesus monkeys. *Food Chem Toxicol.* (2016) 92:165–76. 10.1016/j.fct.2016.04.005 27084109

[56] Kozich J Westcott S Baxter N Highlander S Schloss P. Development of a dual-index sequencing strategy and curation pipeline for analyzing amplicon sequence data on the MiSeq Illumina sequencing platform. *Appl Environ Microbiol.* (2013) 79(17):5112–20. 10.1128/AEM.01043-13 23793624 PMC3753973

[57] Yilmaz P Kottmann R Field D Knight R Cole J Amaral-Zettler L Minimum information about a marker gene sequence (MIMARKS) and minimum information about any (x) sequence (MIxS) specifications. *Nat Biotechnol.* (2011) 29(5):415–20. 21552244 10.1038/nbt.1823 PMC3367316

[58] Bolyen E Rideout J Dillon M Bokulich N Abnet C Al-Ghalith G Reproducible, interactive, scalable and extensible microbiome data science using QIIME 2. *Nat Biotechnol.* (2019) 37(8):852–7. 31341288 10.1038/s41587-019-0209-9 PMC7015180

[59] Martin M. Cutadapt removes adapter sequences from high-throughput sequencing reads. *EMBnet J.* (2011) 17(1):3. 10.1089/cmb.2017.0096 28715235

[60] Callahan B McMurdie P Rosen M Han A Johnson A Holmes S. DADA2: High-resolution sample inference from Illumina amplicon data. *Nat Methods.* (2016) 13(7):581–3. 10.1038/nmeth.3869 27214047 PMC4927377

[61] Robeson M II O’Rourke D Kaehler B Ziemski M Dillon M Foster J RESCRIPt: Reproducible sequence taxonomy reference database management. *PLoS Comput Biol.* (2021) 17(11):e1009581. 10.1371/journal.pcbi.1009581 34748542 PMC8601625

[62] Quast C Pruesse E Yilmaz P Gerken J Schweer T Yarza P The SILVA ribosomal RNA gene database project: Improved data processing and web-based tools. *Nucleic Acids Res.* (2013) 41(Database issue):D590–6. 10.1093/nar/gks1219 23193283 PMC3531112

[63] Pruesse E Quast C Knittel K Fuchs B Ludwig W Peplies J SILVA: A comprehensive online resource for quality checked and aligned ribosomal RNA sequence data compatible with ARB. *Nucleic Acids Res.* (2007) 35(21):7188–96. 10.1093/nar/gkm864 17947321 PMC2175337

[64] Werner J Zhou D Caporaso J Knight R Angenent L. Comparison of Illumina paired-end and single-direction sequencing for microbial 16S rRNA gene amplicon surveys. *Isme J.* (2012) 6(7):1273–6. 10.1038/ismej.2011.186 22170427 PMC3379627

[65] Callahan B McMurdie P Holmes S. Exact sequence variants should replace operational taxonomic units in marker-gene data analysis. *ISME J.* (2017) 11(12):2639–43. 10.1038/ismej.2017.119 28731476 PMC5702726

[66] Bokulich N Kaehler B Rideout J Dillon M Bolyen E Knight R Optimizing taxonomic classification of marker-gene amplicon sequences with QIIME 2’s q2-feature-classifier plugin. *Microbiome.* (2018) 6(1):90. 10.1186/s40168-018-0470-z 29773078 PMC5956843

[67] Janssen S McDonald D Gonzalez A Navas-Molina J Jiang L Xu Z Phylogenetic placement of exact amplicon sequences improves associations with clinical information. *mSystems.* (2018) 3(3):e00021-18. 10.1128/mSystems.00021-18 29719869 PMC5904434

[68] Lin H Peddada S. Analysis of compositions of microbiomes with bias correction. *Nat Commun.* (2020) 11(1):3514. 10.1038/s41467-020-17041-7 PMC7360769 32665548

[69] Fernandes A Macklaim J Linn T Reid G Gloor GB. ANOVA-like differential expression (ALDEx) analysis for mixed population RNA-Seq. *PLoS One.* (2013) 8(7):e67019. 10.1371/journal.pone.0067019 23843979 PMC3699591

[70] *ALDEx2: ANOVA-Like Differential Expression Tool for Compositional Data. [Internet].* n.d. Available online at: https://github.com/ggloor/ALDEx2

[71] Park J Yoon H Lee SB Kim HJ Jung YH Choi CW Do different samples from pregnant women and their neonates share the common microbiome: A prospective cohort study. *Res Square [Preprint].* (2021): 10.21203/rs.3.rs-1062191/v1

[72] Prokopidis K Mazidi M Sankaranarayanan R Tajik B McArdle A Isanejad M. Effects of whey and soy protein supplementation on inflammatory cytokines in older adults: A systematic review and meta-analysis. *Br J Nutr.* (2023) 129(5):759–70. 10.1017/S0007114522001787 35706399 PMC9975787

[73] Metzger C Narayanan S Zawieja D Bloomfield SA. A moderately elevated soy protein diet mitigates inflammatory changes in gut and in bone turnover during chronic TNBS-induced inflammatory bowel disease. *Appl Physiol Nutr Metab.* (2019) 44(6):595–605. 10.1139/apnm-2018-0514 30352170

[74] Kakino S Ohki T Nakayama H Yuan X Otabe S Hashinaga T Pivotal role of TNF-α in the development and progression of nonalcoholic fatty liver disease in a murine model. *Horm Metab Res.* (2018) 50(1):80–7. 10.1055/s-0043-118666 28922680

[75] Wandrer F Liebig S Marhenke S Vogel A John K Manns M TNF-Receptor-1 inhibition reduces liver steatosis, hepatocellular injury and fibrosis in NAFLD mice. *Cell Death Dis.* (2020) 11(3):212. 10.1038/s41419-020-2411-6 32235829 PMC7109108

[76] Li W Hakkak R. Soy protein concentrate diets inversely affect LPS-binding protein expression in colon and liver, reduce liver inflammation, and increase fecal LPS excretion in obese zucker rats. *Nutrients [Internet].* (2024) 16(7):982. 10.3390/nu16070982 38613016 PMC11013665

[77] Kawai T Autieri M Scalia R. Adipose tissue inflammation and metabolic dysfunction in obesity. *Am J Physiol Cell Physiol.* (2021) 320(3):C375–91. 33356944 10.1152/ajpcell.00379.2020 PMC8294624

[78] Albillos A de Gottardi A Rescigno M. The gut-liver axis in liver disease: Pathophysiological basis for therapy. *J Hepatol.* (2020) 72(3):558–77. 31622696 10.1016/j.jhep.2019.10.003

[79] Boutagy N McMillan R Frisard M Hulver M. Metabolic endotoxemia with obesity: Is it real and is it relevant? *Biochimie.* (2016) 124:11–20. 10.1016/j.biochi.2015.06.020 26133659 PMC4695328

[80] Nier A Huber Y Labenz C Michel M Bergheim I Schattenberg J. Adipokines and endotoxemia correlate with hepatic steatosis in non-alcoholic fatty liver disease (NAFLD). *Nutrients.* (2020) 12(3):699. 10.3390/nu12030699 32151020 PMC7146245

[81] Carpino G Del Ben M Pastori D Carnevale R Baratta F Overi D Increased liver localization of lipopolysaccharides in human and experimental NAFLD. *Hepatology.* (2020) 72(2):470–85. 10.1002/hep.31056 31808577

[82] Fukunishi S Sujishi T Takeshita A Ohama H Tsuchimoto Y Asai A Lipopolysaccharides accelerate hepatic steatosis in the development of nonalcoholic fatty liver disease in Zucker rats. *J Clin Biochem Nutr.* (2014) 54(1):39–44. 10.3164/jcbn.13-49 24426189 PMC3882483

[83] He Q Zeng J Yao K Wang W Wu Q Tang R Long-term subcutaneous injection of lipopolysaccharides and high-fat diet induced non-alcoholic fatty liver disease through IKKε/ NF-κB signaling. *Biochem Biophys Res Commun.* (2020) 532(3):362–9. 32883523 10.1016/j.bbrc.2020.08.036

[84] Hakkak R Al-Dwairi A Fuchs G Korourian S Simmen F. Dietary soy protein induces hepatic lipogenic enzyme gene expression while suppressing hepatosteatosis in obese female Zucker rats bearing DMBA-initiated mammary tumors. *Genes Nutr.* (2012) 7(4):549–58. 10.1007/s12263-012-0294-6 22528625 PMC3448032

[85] Kozaczek M Bottje W Kong B Dridi S Albataineh D Lassiter K Long-term soy protein isolate consumption reduces liver steatosis through changes in global transcriptomics in obese zucker rats. *Front Nutr.* (2020) 7:607970. 10.3389/fnut.2020.607970 33363197 PMC7759473

[86] Forbes J Van Domselaar G Bernstein C. The gut microbiota in immune-mediated inflammatory diseases. *Front Microbiol.* (2016) 7:1081. 10.3389/fmicb.2016.01081 27462309 PMC4939298

[87] Zheng D Liwinski T Elinav E. Interaction between microbiota and immunity in health and disease. *Cell Res.* (2020) 30(6):492–506. 32433595 10.1038/s41422-020-0332-7 PMC7264227

[88] Tilg H Zmora N Adolph T Elinav E. The intestinal microbiota fuelling metabolic inflammation. *Nat Rev Immunol.* (2020) 20(1):40–54. 31388093 10.1038/s41577-019-0198-4

[89] Mishra S Wang B Jain S Ding J Rejeski J Furdui C A mechanism by which gut microbiota elevates permeability and inflammation in obese/diabetic mice and human gut. *Gut.* (2023) 72(10):1848–65. 10.1136/gutjnl-2022-327365 36948576 PMC10512000

[90] Portincasa P Bonfrate L Khalil M Angelis M Calabrese F D’Amato M Intestinal barrier and permeability in health, obesity and NAFLD. *Biomedicines.* (2021) 10(1):83. 10.3390/biomedicines10010083 35052763 PMC8773010

[91] Cani P Possemiers S Van de Wiele T Guiot Y Everard A Rottier O Changes in gut microbiota control inflammation in obese mice through a mechanism involving GLP-2-driven improvement of gut permeability. *Gut.* (2009) 58(8):1091. 10.1136/gut.2008.165886 19240062 PMC2702831

[92] Debelius J Huang T Cai Y Ploner A Barrett D Zhou X Subspecies niche specialization in the oral microbiome is associated with nasopharyngeal carcinoma risk. *mSystems.* (2020) 5(4):e00065-20. 10.1128/mSystems.00065-20 32636333 PMC7343305

[93] Smith Byron J Miller Richard A Schmidt Thomas M. Muribaculaceae genomes assembled from metagenomes suggest genetic drivers of differential response to acarbose treatment in mice. *mSphere.* (2021) 6(6):e0085121. 10.1128/msphere.00851-21 34851167 PMC8636109

[94] Zhang M Zhang M Kou G Li Y. The relationship between gut microbiota and inflammatory response, learning and memory in mice by sleep deprivation. *Front Cell Infect Microbiol.* (2023) 13:1159771. 10.3389/fcimb.2023.1159771 37293204 PMC10244646

[95] Bai D Zhao J Wang R Du J Zhou C Gu C Eubacterium coprostanoligenes alleviates chemotherapy-induced intestinal mucositis by enhancing intestinal mucus barrier. *Acta Pharm Sin B.* (2024) 14(4):1677–92. 10.1016/j.apsb.2023.12.015 38572095 PMC10985029

[96] Kenny D Plichta D Shungin D Koppel N Hall A Fu B Cholesterol metabolism by uncultured human gut bacteria influences host cholesterol level. *Cell Host Microbe.* (2020) 28(2):245–57.e6. 10.1016/j.chom.2020.05.013 32544460 PMC7435688

[97] Kriaa A Bourgin M Potiron A Mkaouar H Jablaoui A Gérard P Microbial impact on cholesterol and bile acid metabolism: Current status and future prospects. *J Lipid Res.* (2019) 60(2):323–32. 10.1194/jlr.R088989 30487175 PMC6358303

[98] Bartlett A Kleiner M. Dietary protein and the intestinal microbiota: An understudied relationship. *iScience.* (2022) 25(11):105313. 10.1016/j.isci.2022.105313 PMC9626677 36339270

[99] Wu S Bhat Z Gounder R Mohamed Ahmed I Al-Juhaimi F Ding Y Effect of dietary protein and processing on gut microbiota-A systematic review. *Nutrients.* (2022) 14(3):453. 10.3390/nu14030453 35276812 PMC8840478

[100] Kleiner D Brunt E Van Natta M Behling C Contos M Cummings O Design and validation of a histological scoring system for nonalcoholic fatty liver disease. *Hepatology.* (2005) 41(6):1313–21. 15915461 10.1002/hep.20701

