# Supplementary material for: Dietary soy protein reverses obesity-induced liver steatosis and alters fecal microbial composition independent of isoflavone level
Source: Front Nutr. 2024 Oct 28;11:1487859. doi: 10.3389/fnut.2024.1487859 (PMC11551038; doi:10.3389/fnut.2024.1487859)
Supplement: Supplementary file 1 [file Table_1.DOCX]

Supplementary Table 1. Mean comparisons of casein diet-fed obese Zucker rats at 8 and 18 weeks

|  |  | Casein (8-weeks) | Casein (16-weeks) | p-value |
| --- | --- | --- | --- | --- |
|  |  |  |  |  |
| Body weight (BW) |  | 615.4 (25.0) | 679.4 (57.9) | 0.043 |
|  |  |  |  |  |
| Liver weight (% of BW) |  | 6.7 ( 1.2) | 6.9 (0.9) | 0.703 |
|  |  |  |  |  |
| ALT |  | 80.7 (10.7) | 43.3 (16.4) | 0.003 |
|  |  |  |  |  |
| AST |  | 267.5 (48.9) | 276.7 (84.4) | 0.949 |
|  |  |  |  |  |
| Steatosis score |  | 3.1 (0.7) | 2.7 (1.0) | 0.354 |
|  |  |  |  |  |
| Microvesicular score |  | 3.1 (0.7) | 2.3 (1.0) | 0.078 |
|  |  |  |  |  |
| Macrovesicular score |  | 1.6 (0.5) | 1.4 (0.8) | 0.698 |

ALT - alanine transaminase; AST – aspartate transaminase.
